# Influenza A(H1N1)pdm09 and Cystic Fibrosis Lung Disease: A Systematic Meta-Analysis

**DOI:** 10.1371/journal.pone.0078583

**Published:** 2014-01-10

**Authors:** Hanna Renk, Nicolas Regamey, Dominik Hartl

**Affiliations:** 1 University Children's Hospital, Eberhard-Karls-University, Tuebingen, Germany; 2 Department of Paediatrics, Inselspital and University of Bern, Bern, Switzerland; University Hospital Freiburg, Germany

## Abstract

**Background:**

To systematically assess the literature published on the clinical impact of Influenza A(H1N1)pdm09 on cystic fibrosis (CF) patients.

**Methods:**

An online search in PUBMED database was conducted. Original articles on CF patients with Influenza A(H1N1)pdm09 infection were included. We analyzed incidence, symptoms, clinical course and treatment.

**Results:**

Four surveys with a total of 202 CF patients infected by Influenza A(H1N1)pdm09 were included. The meta-analysis showed that hospitalisation rates were higher in CF patients compared to the general population. While general disease symptoms were comparable, the clinical course was more severe and case fatality rate (CFR) was higher in CF patients compared to asthmatics and the general population.

**Conclusions:**

Evidence so far suggests that CF patients infected with Influenza A(H1N1)pdm09 show increased morbidity and a higher CFR compared to patients with other chronic respiratory diseases and healthy controls. Particularly, CF patients with advanced stage disease seem to be more susceptible to severe lung disease. Accordingly, early antiviral and antibiotic treatment strategies are essential in CF patients. Preventive measures, including vaccination as well as hygiene measures during the influenza season, should be reinforced and improved in CF patients.

## Introduction

### Influenza A (H1N1)pdm09

A new Influenza A(H1N1) virus subtype with a large antigenic difference to the previously encountered seasonal A(H1N1) viruses was detected for the first time in Mexico in April 2009 [1]. The virus rapidly spread around the world and in June 2009 the first influenza pandemic of the twenty-first century was declared [2]. Today, in the post-pandemic period, the Influenza A(H1N1)pdm09 virus has adopted the properties of a seasonal influenza virus [3]. Several countries have already experienced seasonal outbreaks during the last few years, but since the Influenza A(H1N1)pdm09 virus has now become one of the seasonal strains of influenza viruses it has lost public interest [4]–[7]. However, at the beginning of 2013, the Influenza A(H1N1)pdm09 virus launched a remarkable “comeback” in some regions like the Middle East, Northern Africa and some European countries, e.g. Germany. Influenza A(H1N1)pdm09 was the predominant laboratory confirmed subtype in Europe in the 2013 winter season [8].

Influenza A(H1N1)pdm09, first isolated in 2009, is a reassortment between four different viruses: North American swine influenza, Eurasian swine influenza, avian influenza and human influenza. The virus is transmitted by direct contact with infected individuals, contaminated objects or by inhalation of virus-laden aerosols. The incubation period is 1.5–3 days. Influenza A(H1N1)pdm09 differs in pathogenic mechanisms, epidemiological features and virulence from previous seasonal influenza A viruses. In contrast to other influenza viruses, the pdm09 subtype lacks mutations which are responsible for high pathogenicity. Replication of the virus takes longer and the level of pulmonary replication is higher than in other seasonal influenza viruses [9], [10].

Incidence rates of Influenza A(H1N1)pdm09 in adults and children with influenza-like illnesses (ILI) were reported to be 33% and 36%, respectively during the pandemic [11], [12]. Contrary to expectations during the pandemic, Influenza A(H1N1)pdm09 caused only relatively mild disease in the general population. Main symptoms of infection were fever and cough in around 80% of patients, followed by dyspnea, fatigue and myalgias, headache, sore throat and gastrointestinal symptoms [13]. Hospitalization rates ranged between 7–20% at the beginning of the pandemic [14]. Younger age groups, including patients with chronic respiratory diseases such as CF patients, were particularly affected by the Influenza A(H1N1)pdm09 virus during the 2009 pandemic. In seasonal influenza (eg H3N2 and H1N1) hospitalization rates among children and adults aged between 18 and 64 years were lower than in Influenza A(H1N1)pdm09. Vice versa, among elderly persons hospitalization rates were lower in Influenza A(H1N1)pdm09 than in seasonal influenza strains [15]. 90% of all deaths occurred in persons <65 years and the highest hospitalization rates were found in children <5 years of age [16], [17]. Ethnic minorities and children with pre-existing disorders were disproportionately affected [17]. Risk factors for severe course included age <5 years or ≥65 years, obesity, underlying neurological diseases, diabetes, cardiovascular disease, chronic kidney or liver disease, immunosuppression and/or pregnancy [18], [19]. In a large observational study in England during the pandemic period, overall childhood mortality rate was 6 per million population. Children with severe pre-existing disorders such as chronic neurological, gastrointestinal or respiratory disease accounted for 64% of deaths [17].

Bacterial coinfection was the cause of death of many individuals with Influenza A(H1N1)pdm09. In lung tissue specimens from fatal cases, bacterial coinfection occurred in 22/77 (29%) patients [20]. Furthermore in a prospective Canadian study, bacterial coinfection was confirmed by positive culture in 259/681 (38%) of adult ICU admissions [21]. The most common pathogens isolated from respiratory cultures in critically ill children with Influenza A(H1N1)pdm09 were *Staphylococcus aureus* (including *MRSA*) (39%), *Pseudomonas species* (16%), *Streptococcus pneumoniae* (8%), *Haemophilus influenzae* (7%) and *Streptococcus pyogenes* (4%) [22]. Thus, secondary bacterial infection is a leading cause of severe course and death in Influenza A(H1N1)pdm09 virus infection. Influenza A(H1N1)pdm09 related death was reported in 7% and 5.3% of patients hospitalized with ILI and PCR confirmed infection [18], [19]. Case fatality rate (CFR) of Influenza A(H1N1)pdm09 infection in the general population was overestimated at the beginning of the pandemic and finally ranged 2.9 to 7-fold lower than in seasonal influenza infections from 1990–1999 [23]. Contrarily to the early expectations, CFR of Influenza A(H1N1)pdm09 infection was retrospectively calculated only around 0.04% to 0.05% [24], [25].

### Cystic fibrosis

Cystic fibrosis (CF) is the most common autosomal recessive inherited lethal metabolic disorder of the white population with an incidence of about 1 in 2000–3000 live births [26], [27]. The disease is caused by a mutation in the *cystic fibrosis transmembrane conductance regulator gene (CFTR)* resulting in a dysfunctional protein of the apical membrane of epithelial cells [28]. This protein serves as a cAMP-regulated chloride channel. Lack or dysfunction leads to ineffective chloride secretion and increased absorption of water from the respiratory tract. Consequently, mucociliary clearance is impaired and viscous mucus is present, which in turn favors chronic bacterial colonization of the airways and pulmonary inflammation. Chronic pulmonary disease accounts for most of the morbidity and mortality in CF. Airway obstruction, infection with typical pathogens including *Staphylococcus aureus*, *Haemophilus influenza* and *Pseudomonas aeruginosa*, and inflammation lead to progressive deterioration of pulmonary function [29]. Cough, increased sputum production and dyspnea are the most prominent signs of pulmonary manifestations of the disease.

Epithelial cells also are affected by reduced or defective *CFTR* activity in other organs, which leads to mucinous obstruction of various secretory glands. Pancreatic insufficiency, intestinal obstruction, liver damage and infertility may occur. Since the identification of the *CFTR* gene in 1989, the life expectancy of CF patients has continuously increased, due to improved treatment. Nowadays, the average life expectancy of CF patients in countries of the western world adds up to 40 years [30].

Recently, viral respiratory infections in CF were given more and more attention [31], [32]. In pediatric and adult CF patients the risk of pulmonary exacerbations as well as length and frequency of hospitalizations were found to be increased by viral respiratory infections. Additionally, viral respiratory infections in CF patients have been shown to be associated with deterioration of pulmonary function and predispose to secondary bacterial infection [33]–[35]. It does not seem that CF patients are affected more frequently by viral respiratory infections than the general population, but the clinical consequences are worse. The rate of symptomatic respiratory illnesses was significantly higher in CF patients than in their healthy siblings [36]. It was also shown that children with CF were affected longer and more severely by viral respiratory disease [37]. Viral respiratory infection in general and especially RSV and Influenza might have serious consequences for CF patients [38].

Currently, little is known about short-term morbidity and disease progression associated with recently detectable viruses such as newly recognized rhinovirus subtypes, human metapneumovirus (hMPV) and coronaviruses. In particular, the impact of the Influenza A(H1N1)pdm09 virus on CF patients is unclear. In this study, we aimed at describing the impact of the Influenza A(H1N1)pdm09 pandemic on CF patients based on a retrospective data analysis of the currently available literature. We wished to answer the following questions: What impact did the Influenza A(H1N1)pdm09 pandemic have on the CF population and what impact of the Influenza A(H1N1)pdm09 virus has to be expected in the future for CF patients? What recommendations for prevention and treatment of CF patients with Influenza A(H1N1)pdm09 infection can be given according to the current knowledge?

## Methods

We performed a systematic literature search in PUBMED for original articles from 2009 to 2013, describing the effects of Influenza A(H1N1)pdm09 infection on CF patients. All published articles since the onset of the Influenza A (H1N1)pdm09 pandemic in April 2009 were extracted. The search term was “cystic fibrosis [MeSH] and H1N1 influenza”, generating 16 results. Case reports, non-clinical studies and publications dealing just with H1N1 vaccination or pharmacokinetics of Oseltamivir were excluded. The search yielded four original articles from four different countries, all published in English language. Two surveys had been conducted as multicenter studies. All studies were designed retrospectively and data collection was based on questionnaires as well as web-based in the two multicenter studies. Incidence (i.e. incidence of the illness), symptoms, treatment and clinical course (i.e. hospitalization, ICU treatment, death) of Influenza A (H1N1)pdm09 infection in CF patients were analyzed from 06/2009 to 04/2010. Out of the four studies and their supplements the following data were recorded: name of first author, journal, country and year of publication, period of analysis, number, age and characteristics of Influenza A(H1N1)pdm09 positive CF patients (eg *Pseudomonas* colonization, percent predicted forced expiratory volume in one second (FEV1% pred.), vaccination status), symptoms associated with Influenza A(H1N1)pdm09 infection, treatment and clinical course. Mean values of the frequency of clinical symptoms in the different studies were calculated. Similar symptom groups were summarized.

Clinical course was not described consistently in the four studies, therefore “mild course” was defined as outpatient treatment, “moderate course” as hospitalization without complications and “serious course” as intensive care treatment, mechanical ventilation, or death due to complications. In addition, conclusions and recommendations of the authors regarding Influenza A(H1N1)pdm09 infection in CF patients were summarized.

### Results of the literature review

The four studies included in the meta-analysis are summarized in Table 1. Two studies were performed as multicenter surveys, one Europe-wide, the other one confined to Italy. The two single-center studies were conducted in England and Australia. A total of 5429 subjects with CF were included in the four studies, and Influenza A (H1N1)pdm09 infection was confirmed by PCR in 202 of them (3.7%). The European study by Viviani et al. was the largest, including 110 patients [39].

**Table 1:** Characteristics, treatment and outcome of Influenza A(H1N1)pdm09-infected CF patients.

Country	Inf A(H1N1)pdm09 infection in CF patients			Characteristics of CF patients infected with Inf A(H1N1)pdm09 (%)				Treatment (%)	Outcome (%)			Conclusion/Recommendation	Ref
	*N* 1	*Age* 2	*Incidence* * *(%)*	*P. aerug.*	*Pancreatic*	*FEV1*	*Vacc*	*Oseltamivir*	*Hospital*	*ICU* 4 *or*	*CFR* 5		
				*//*	*Insufficiency*	*pred* 3		*//*	*admission*	*ventilation*			
				*B. cepacia*				*Antibiotics*					
				*coinfection*									
Europe#	110	13	2.3*	n.r.	84	69	8.8	81/66	48	5.4	2.7	awareness,	[39]
								81//66					
												infection control,	
												vaccination campaign	
Italy#	68	15	53**	48.5//8.8	89.7	53	13.2	82//68	69	1.5 ventilation	4.4	annual vaccination in	[41]
				48.5//8.8									
										13.2 on oxygen		adult CF patients	
Australia	11	22	4.4*	100//1	n.r.	66	n.av.	100//75	63	n.r.	n.r.	prompt	[42]
				100//1									
												commencement of	
												oseltamivir and	
												antibiotic therapy	
UK	13	22	4*	100//n.r.	n.r.	51.4	n.r.	100//100	69	0	n.r.	generally mild illness	[40]
					n.r.			100//100					
												in CF,	
												potentially more	
												virulent pandemic in	
												the future	

^1^ Number of Influenza A(H1N1)pdm09 positive CF patients.

^2^ Mean age (years).

^3^ Mean of all CF patients studied.

^4^ Intensive care unit.

^5^ Case fatality rate.

^#^ multicenter surveys;

incidence of Inf A(H1N1)pdm09 infections in CF patients.

incidence of Inf A(H1N1)pdm09 infections relating to CF patients with influenza-like illness.

(ILI); n.r. – not reported; Vacc – Vaccination coverage; P. aerug. = Pseudomonas aeruginosa; B. cepacia = Burkholderia cepacia.

#### Incidence

attributable to Influenza A(H1N1)pdm09 ranged from 2.3% in the Europe-wide multicenter study (0% to 9.4% depending on the center) to 4.4% in the Australian study by Nash et al. [40]. In the Italian study by Colombo et al., the proportion of patients who were tested positive for Influenza A(H1N1)pdm09 (53%) was based only on CF patients with ILI, and therefore incidence was not calculable [41].

#### Patient characteristics

The mean age of the 202 Influenza A(H1N1)pdm09-positive patients ranged from 13 to 22 years in the four studies. The majority of the patients were infected chronically with *Pseudomonas aeruginosa*. In two studies, a rate of pancreatic insufficiency of 85–90% was reported. The pulmonary status was described by mean FEV1% pred. in all studies and ranged between 51% and 69%. Due to the lack of general availability of the Influenza A(H1N1)pdm09 vaccine until the end of 2009, immunization data of Influenza A(H1N1)pdm09 vaccination were not documented in the Australian and the English study. The two multicenter studies reported an Influenza A(H1N1) vaccination rate of 8.8% and 13.2%, respectively in CF patients.

#### Symptomatology

**Figure 1:**
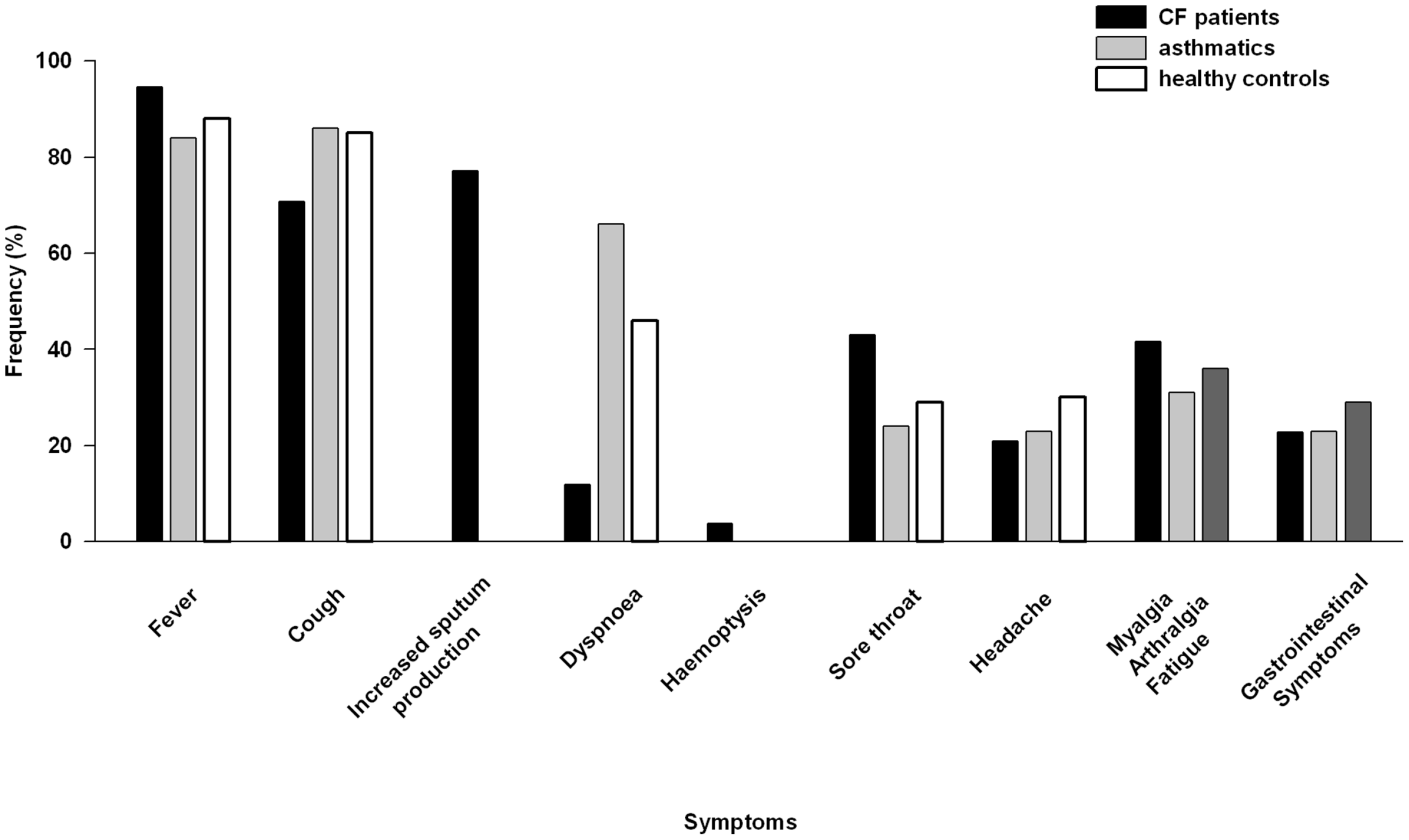
Symptoms of Influenza A(H1N1)pdm09 infection in CF, asthmatics and healthy controls. Frequency of presenting symptoms in CF patients, asthmatics and healthy controls from four studies of Influenza A(H1N1)pdm09 infection in CF patients [39]–[42], and a study of asthmatics and healthy controls [13] during the 2009 pandemic.

Fever occurred in nearly all cases (90–100%), increased cough and sputum production were consistently the most common symptoms. Dyspnea, sore throat, headache, myalgia, arthralgia, fatigue and gastrointestinal symptoms occurred in only 50% of cases or less. Rarely hemoptysis (in 4.5%) was reported. In Figure 1, each symptom is compared with frequency of symptoms in asthmatics and subjects with no comorbidities infected by Influenza A(H1N1)pdm09 from a US study by McKenna [13].

#### Treatment

In all studies Oseltamivir was used exclusively for antiviral therapy. In the two multicenter trials approximately 80% of patients were treated with Oseltamivir and antibiotic treatment was administered in 66% and 68%, respectively. In the two other studies, all patients received Oseltamivir and antibiotics were given in 75% and 100% of cases, respectively.

#### Clinical course

**Figure 2:**
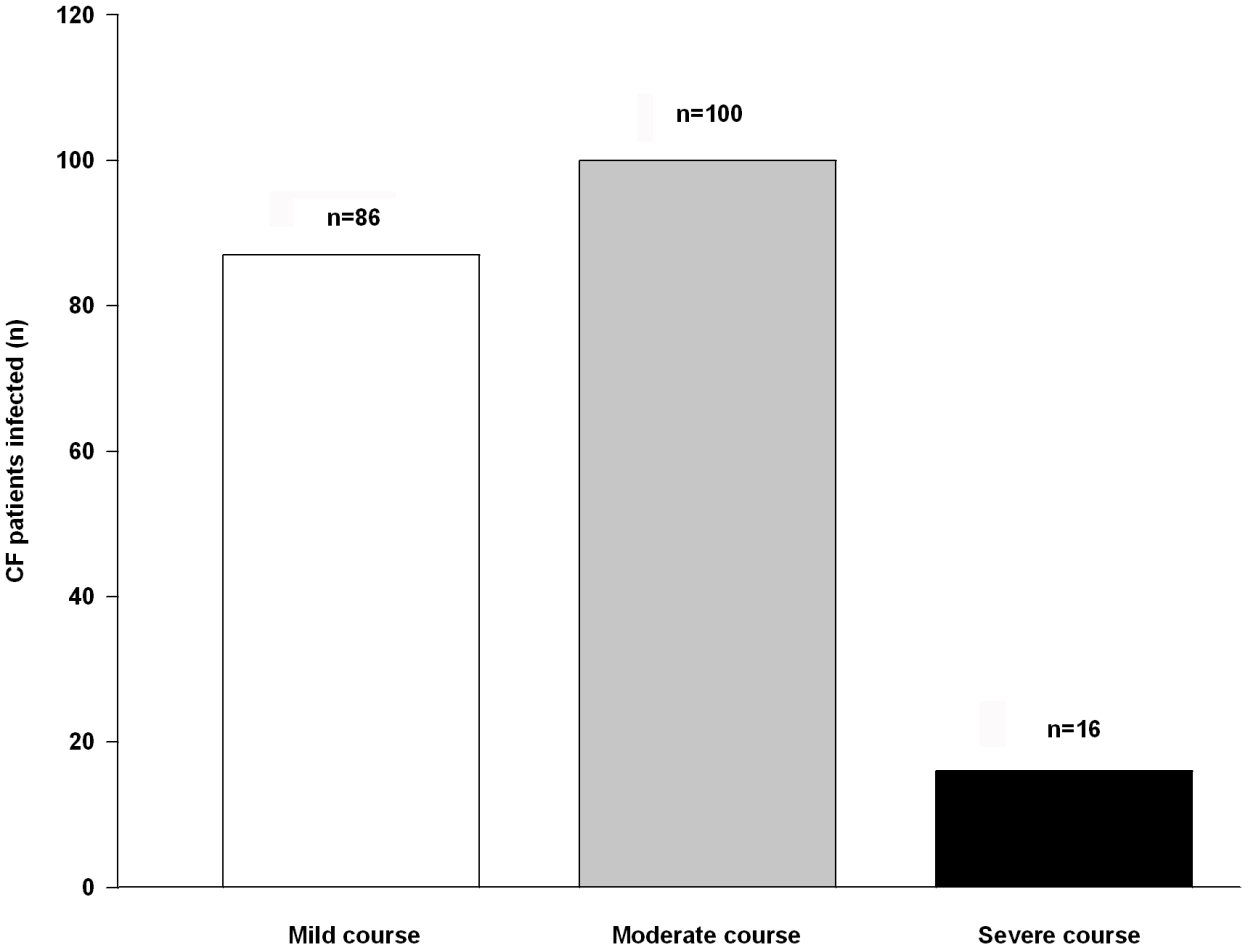
Clinical course of Influenza A(H1N1)pdm09 infection in CF patients. Number of CF patients with mild, moderate or severe course of Influenza A(H1N1)pdm09 infection in the analyzed studies [39]–[42]. Mild course: outpatient treatment. Moderate course: hospitalization without complications. Serious course: intensive care treatment, mechanical ventilation, or death.

There was no consistent description of the clinical course of Influenza A(H1N1)pdm09 infection in CF patients in the four studies. Mild course (outpatient treatment) occurred in 86/202 (43%); moderate course (hospitalization without complications) in 100/202 (49%) and severe course (intensive care treatment or death) in 16/202 (8%) (Figure 2). Mortality rates (2.7% and 4.4%, respectively) were available only from the two multicenter trials.

#### Recommendations and conclusions

from the four studies are summarized in headwords in Table 1. Principal points of prevention and treatment of Influenza A(H1N1)pdm09 infection in CF patients mentioned by the authors included vaccination, infection control measures and the rapid onset of aggressive antiviral and antibiotic treatment.

## Discussion

Our search revealed that, surprisingly, there are only few data on CF patients infected with Influenza A(H1N1)pdm09 in the literature. Merely four studies could be identified, and data were only available from the pandemic in 2009/2010.

### Incidence

Incidence of Influenza A(H1N1)pdm09 in the four studies was not consistently described. Results of the multicenter Europe-wide survey of Viviani et al. [39] and the studies of Nash et al. [40] and France et al. [42] refer to the total CF population, regardless of symptoms of ILI. Incidence of Influenza A(H1N1)pdm09 infection ranged from 2.3 to 4.4% in these studies. However, Colombo et al. specifically referred to CF patients with ILI and 68/127 (53%) patients were tested positive for Influenza A(H1N1)pdm09 [41]. This rate is remarkably increased compared to the general population with ILI, where 36% of patients in a pediatric and 34% in an adult emergency room were proven positive during the pandemic [11], [12]. Pecavar et al. used a similar study period and case definition of ILI compared to Colombo's data in italian CF patients [11], [41]. Although local differences, like a higher amount of circulating virus in the Italian centers, is a possible reason for the difference, these data suggest that CF patients may have an increased susceptibility to the Influenza A(H1N1)pdm09 virus, at least during the pandemic period. However, to verify this, additional evidence needs to be collected during future influenza seasons.

### Patient characteristics

Particularly CF patients in advanced stages of the disease seem susceptible to Influenza A(H1N1)pdm09 infection. The mean of FEV1% pred., which is a prognostic factor for progression of pulmonary disease, was poor (50 to 70%) in the analyzed studies. On average, patients aged up to 22 years have a higher FEV1% pred. [43]. Consequently, reported CF patients infected by Influenza A(H1N1)pdm09, often had poor pulmonary conditions. One may conclude that patients in advanced stages of CF suffer the most from increased Influenza A(H1N1)pdm09 mediated incidence, like it has already been described for viral respiratory infections in CF patients in general [36]. However, it is possible that only centers with severely affected patients reported their cases and thus reporting bias also provides an explanation for the increased incidence.

### Symptomatology

The clinical presentation of Influenza A(H1N1)pdm09 infection differed only slightly from the symptomatology in the general population and from asthmatics. The main symptom of infection was fever in 92–100%, followed by cough, sore throat and other symptoms characteristic for common viral respiratory infections. However, increased sputum production was found to predominate and rarely hemoptysis was present in CF patients with Influenza A(H1N1)pdm09 compared to the general population and asthmatics [13], [18].

### Treatment

Early antiviral therapy and, if appropriate, antibiotic coverage in CF patients are crucial for the treatment of Influenza A(H1N1)pdm09 infection. Antiviral treatment with Oseltamivir was performed in all of the analyzed studies. Early start of treatment during the first three days of disease reduces viral shedding and thus is not only of therapeutic use, but also contributes to infection control [44].

Antibiotics were administered in 66–100% of infected CF patients. During former and during the 2009 influenza pandemic, bacterial coinfection caused substantial morbidity and mortality in healthy individuals and in patients with comorbidities [45], [46]. In this context, CF patients, frequently colonized with *Pseudomonas aeruginosa* and other pathogens, seem to be at high risk for pulmonary exacerbation and fatal course from bacterial coinfection during an influenza outbreak. Therefore, adequate initiation of appropriate antibiotic treatment, like it was done in the analyzed studies, is important. However, currently there is no consensus of optimal mode of antibiotic therapy (single or combination) as well as its duration in CF patients [47].

### Clinical course

Approximately 50–70% of all CF patients tested positive required hospitalization. This high rate in CF patients compared to the general population (hospitalization rate 7–20%) should be interpreted with caution, though [13]. For medical staff confronted with CF patients experiencing a H1N1 infection, the need for hospitalization was difficult to estimate at the beginning of the pandemic phase due to lack of experience with the clinical course of the disease. This likely resulted in excess hospitalizations, especially in patients with comorbidities. Therefore, the high hospitalization rate in CF patients may in fact not correspond to a more severe course of the disease.

In general, patients with comorbidities, especially chronic respiratory diseases, were at particular risk for severe course and complications of Influenza A(H1N1)pdm09 infection [19]. Collectively, clinical course in the four studies was worse in CF patients compared to the general population. More than half of all CF patients (57%) showed a moderate or severe course of disease, requiring hospitalization or even ICU treatment (Figure 2). These data are consistent with previous studies, reporting a three-fold increase for ICU admission or fatal course in patients with chronic lung disease (other than asthma) infected by Influenza A(H1N1)pdm09 [18], [19]. Accordingly, CF lung disease, in contrast to asthma, is likely to represent a clinical risk factor for severe disease course in Influenza A(H1N1)pdm09 infection [48]. A possible explanation for this might be that CF patients generally do not suffer more often from viral infections than non-CF individuals, but with worse clinical consequences. Thus CF patients with viral respiratory infections are prone to exacerbations and severe course [36], [37], [49], [50].

Consistently, the case fatality rate of the two multicenter trials of Viviani et al. and Colombo et al. was increased. A total of 6/4825 CF patients died from Influenza A(H1N1)pdm09 infection, representing a case fatality rate (CFR) of 3.5% (2.7% and 4.4% in each study) [39], [41]. Assuming that this CFR reflects the population related mortality rate of CF patients infected by Influenza A(H1N1)pdm09, it is higher than the CFR of 0.05% in the general population [17], [25].

### Pathophysiological mechanisms

Explaining the increased respiratory morbidity in CF patients towards viral infection are unclear. Several mechanisms have been proposed.

First an alteration of the inflammatory response of CF airway epithelium on virus infection compared to healthy controls has been discussed. On the one hand, increased inflammatory response of CF airway epithelial cells was shown with parainfluenza virus 3 and influenza A infection [51], [52]. CF airway epithelial cells of children also showed a greater IL-8 neutrophilic response after exposure to human rhinovirus than airway epithelial cells of healthy controls in vitro [53]. Thus dysregulation in the innate response of CF airway epithelial cells upon viral infection is possible. Airway inflammation, slower clearance of virus, increased severity and prolonged inflammation may be the consequence of an exaggerated immune response [53]. On the other hand, in a recent study, rhinovirus infection results were vice versa. A lower inflammatory response and increased cell death were found in rhinovirus infected CF airway epithelium, suggesting a greater susceptibility of CF airway epithelium towards viral toxic effects [54].

Second, impaired control of viral replication provides a possible mechanism for increased respiratory morbidity of CF patients. Replication of human rhinovirus (serotype 14 and 1b) and human parainfluenzavirus was increased in airway epithelial cells of CF children compared to control subjects [51], [53]. In influenza infection antiviral response was compromised and apoptotic response reduced. This may lead to higher viral titers as well as a longer time of viral residence and proliferation. Increased viral replication in airway epithelial cells may facilitate a more severe course of viral respiratory illness in CF patients.

A possible explanation is that specific antiviral host defense mechanisms may be compromised by viral respiratory infection. Impairment of antiviral pathways mediated by high–level NO synthesis and IFN/STAT 1 suggests that airway epithelial cells in CF may allow increased viral replication and increased production of proinflammatory cytokines compared to healthy subjects. This results in a higher degree of airway inflammation and severe respiratory symptoms as it was shown for human parainfluenzavirus infection in CF children [51]. In BAL specimens of CF children with rhinovirus infection, a 100 fold higher viral load was detected compared to healthy children. Higher viral load was negatively associated with production of antiviral mediators. Possibly, impaired production of antiviral mediators in CF patients leads to a high viral burden in the lower airways and may particularly occur in the context of CF exacerbations [50].

Finally, viral respiratory infection may trigger pulmonary exacerbation by interaction with bacterial pathogens. In vitro, RSV serves as a coupling agent between airway epithelial cells and *P. aeruginosa* and enhances its adherence to these cells. Thereby, RSV facilitates bacterial colonization (eg with *P. aeruginosa*) [55]. Moreover there is evidence that viral superinfection leads to oxidative stress, followed by dispersal of planktonic bacteria from biofilm matrix. Planktonic bacteria may interact with basolateral receptors in the airway epithelium and stimulate an intense inflammatory response through increased chemokine responses [56].

### Prevention and recommendations

Especially patients in advanced stages of CF, who often have to visit health care facilities, are prone to an Influenza A(H1N1)pdm09 infection with severe course. Therefore, infection control in facilities that care for CF patients is of particular importance. The two main pillars of prevention are vaccination and improvement of hygiene measures. Data on effectiveness of the vaccine in CF patients are scarce and specific immune responses to influenza vaccination in CF patients with advanced disease are not known. Nevertheless, vaccination is strongly recommended by health authorities throughout the world. On the one hand, coverage with the monovalent pandemic vaccine in CF patients was reported to be high but differed markedly between countries [57]. On the other hand, the clinical problem is that the overall acceptance of seasonal influenza vaccination particularly in healthcare workers is poor [58], [59]. In the face of future influenza seasons, efforts to increase vaccination rates in healthcare personnel and close contacts are crucial. Thereby, incidence and serious complications in CF patients might be reduced through herd immunity. Particular attention should be paid on the implementation of hygiene measures in public and private life as well as in hospital settings. Information of CF patients on hygiene and infection control measures e.g. in advance to the influenza season should be performed and combined with influenza vaccination. Furthermore, pneumococcal vaccine provides protection for CF patients from secondary bacterial infection due to *Streptococcus pneumoniae*.

### Limitations of this meta-analysis

This meta-analysis is subject to several limitations. First, there is a certain bias and limited comparability of data due to different methods of analysis and retrospective design of the studies. Second, reporting bias has to be considered in all four surveys. Particularly centers that experienced only mild cases were possibly less likely to participate in the study than those who experienced severe cases. Physicians' awareness and reporting behavior may have influenced data collection and was not assessed. Furthermore different local situations and heterogeneity of the CF population (e.g. children or young adults, different stages of disease) bear further bias. Another caveat relates to the symptoms of disease, which were not consistently described. There is also a lack of systematic assessment of disease severity, which may affect comparability of data. Additionally, calculation of the CFR in the CF population analyzed in these four studies certainly bears a higher inaccuracy than calculations in the general population. However, as diagnosis of Influenza A(H1N1)pdm09 infection was verified by PCR in all studies, a high reliability of the viral detection data and thus a distinct case definition can be assumed.

## Summary

Influenza A(H1N1)pdm09 infection had substantial effects on CF patients during the 2009 pandemic. CF patients seemed to have an increased susceptibility to the virus compared to the general population. Initial clinical symptoms of Influenza A(H1N1)pdm09 infection in CF patients were similar to the general population, beside a significantly increased sputum production. Particularly, patients in advanced stages of CF lung disease were prone to severe course of disease (e.g. ICU admission or death). Early administration of antiviral treatment and antibiotic coverage are crucial. Vaccination and infection control measures should be of public interest at the beginning of an influenza season. However, the impact of Influenza A(H1N1)pdm09 on the CF population in the post-pandemic period remains poorly understood.

## Supporting Information

Checklist S1 **PRISMA Checklist.**
(DOC)
