# Correction: Influenza A(H1N1)pdm09 and Cystic Fibrosis Lung Disease: A Systematic Meta-Analysis

**DOI:** 10.1371/journal.pone.0093142

**Published:** 2014-03-26

**Authors:** The PLOS ONE Staff

In Figure 1, “Symptoms of Influenza A(H1N1)pdm09 infection in CF, asthmatics and healthy controls,” some of the bars are incorrectly shaded. Please see the corrected Figure 1 here.

**Figure 1:**
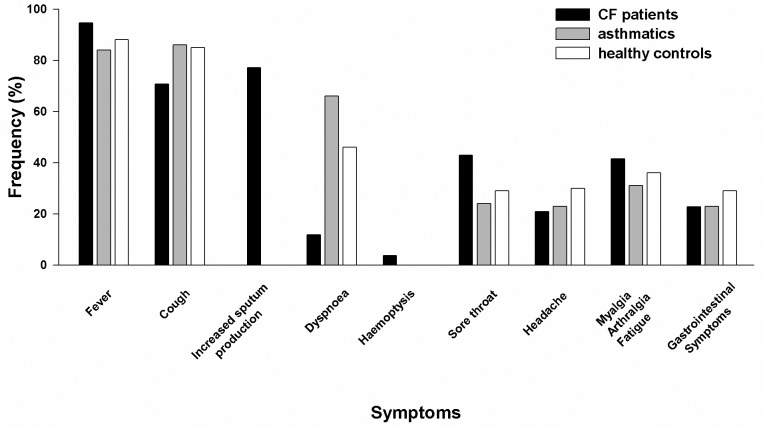
Symptoms of Influenza A(H1N1)pdm09 infection in CF, asthmatics and healthy controls. Frequency of presenting symptoms in CF patients, asthmatics and healthy controls from four studies of Influenza A(H1N1)pdm09 infection in CF patients [39]–[42], and a study of asthmatics and healthy controls [13] during the 2009 pandemic.

Table 1 has been corrected for improved readability. Please see the corrected Table 1 here.

**Table 1:** Characteristics, treatment and outcome of Influenza A(H1N1)pdm09-infected CF patients.

Country	Inf A(H1N1)pdm09 infection in CF patients			Characteristics of CF patients infected with Inf A(H1N1)pdm09 (%)				Treatment (%)	Outcome (%)			Conclusion / Recommendation	Ref
	***N*** 1	***Age*** 2	Incidence* (%)	***P. aerug.*** ***//*** ***B. cepacia coinfection***	***Pancreatic*** ***Insufficiency***	***FEV1 pred^3^***	***Vacc***	***Oseltamivir*** ***//*** ***Antibiotics***	***Hospital admission***	***ICU*** 4 ***or ventilation***	***CFR*** 5		
Europe#	110	13	2.3*	n.r.	84	69	8.8	81 // 66	48	5.4	2.7	awareness, infection control, vaccination campaign	[39]
Italy#	68	15	53**	48.5 // 8.8	89.7	53	13.2	82 // 68	69	1.5 ventilation 13.2 on oxygen	4.4	annual vaccination in adult CF patients	[41]
Australia	11	22	4.4*	100 // 1	n.r.	66	n.av.	100 // 75	63	n.r.	n.r.	prompt commencement of oseltamivir and antibiotic therapy	[42]
UK	13	22	4*	100 // n.r.	n.r.	51.4	n.r.	100 // 100	69	0	n.r.	generally mild illness in CF, potentially more virulent pandemic in the future	[40]

1 Number of Influenza A(H1N1)pdm09 positive CF patients

2 Mean age (years)

3 Mean of all CF patients studied

4 Intensive care unit

5 Case fatality rate

# multicenter surveys; *incidence of Inf A(H1N1)pdm09 infections in CF patients **incidence of Inf A(H1N1)pdm09 infections relating to CF patients with influenza-like illness (ILI); n.r. – not reported; Vacc – Vaccination coverage; P. aerug.  =  *Pseudomonas aeruginosa;* B. cepacia * =  Burkholderia cepacia*.
